# (2*E*)-3-(1,3-Diphenyl-1*H*-pyrazol-4-yl)-1-phenyl­prop-2-en-1-one

**DOI:** 10.1107/S1600536811023282

**Published:** 2011-06-18

**Authors:** Hoong-Kun Fun, Madhukar Hemamalini, Shridhar Malladi, Pradeep Poojari, Arun M Isloor

**Affiliations:** a X-ray Crystallography Unit, School of Physics, Universiti Sains Malaysia, 11800 USM, Penang, Malaysia; b Department of Chemistry, National Institute of Technology, Karnataka, Surathkal, Mangalore 575 025, India

## Abstract

In the title compound, C_24_H_18_N_2_O, the pyrazole ring is essentially planar [maximum deviation = 0.004 (1) Å] and makes dihedral angles of 18.07 (4), 48.60 (4) and 9.13 (5)° with the phenyl rings. In the crystal, adjacent mol­ecules are connected *via* inter­molecular C—H⋯O hydrogen bonds, forming dimers. Furthermore, the crystal structure is stabilized by weak C—H⋯π and π–π inter­actions, with centroid–centroid distances of 3.6808 (5) Å.

## Related literature

For applications of pyrazoles, see: Patel *et al.* (2004 ▶); Isloor *et al.* (2009 ▶); Vijesh *et al.* (2010 ▶); Sharma *et al.* (2010 ▶); Rostom *et al.* (2003 ▶); Ghorab *et al.* (2010 ▶); Amnekar & Bhusari (2010 ▶). For the synthetic procedure, see: Sharma *et al.* (2010 ▶). For the stability of the temperature controller used in the data collection, see: Cosier & Glazer (1986 ▶).
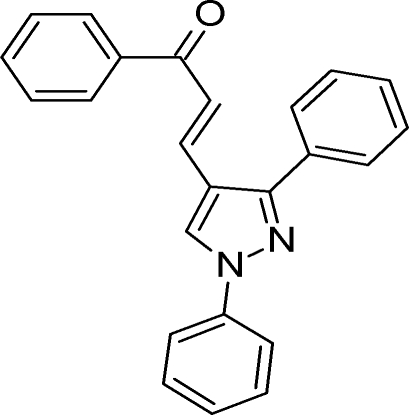


## Experimental

### 

#### Crystal data

C_24_H_18_N_2_O
*M* *_r_* = 350.40
Triclinic, 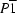

*a* = 8.1027 (2) Å
*b* = 9.3157 (2) Å
*c* = 12.9634 (3) Å
α = 73.630 (1)°
β = 74.713 (1)°
γ = 74.820 (1)°
*V* = 886.83 (4) Å^3^
*Z* = 2
Mo *K*α radiation
μ = 0.08 mm^−1^
*T* = 100 K
0.66 × 0.23 × 0.16 mm

#### Data collection

Bruker SMART APEXII CCD area-detector diffractometer
Absorption correction: multi-scan (*SADABS*; Bruker, 2009 ▶) *T*_min_ = 0.949, *T*_max_ = 0.988
27273 measured reflections
7371 independent reflections
6190 reflections with *I* > 2σ(*I*)
*R*_int_ = 0.024

#### Refinement

*R*[*F* ^2^ > 2σ(*F* ^2^)] = 0.044
*wR*(*F* ^2^) = 0.131
*S* = 1.04
7371 reflections
244 parameters
H-atom parameters constrained
Δρ_max_ = 0.50 e Å^−3^
Δρ_min_ = −0.30 e Å^−3^

### 

Data collection: *APEX2* (Bruker, 2009 ▶); cell refinement: *SAINT* (Bruker, 2009 ▶); data reduction: *SAINT*; program(s) used to solve structure: *SHELXTL* (Sheldrick, 2008 ▶); program(s) used to refine structure: *SHELXTL*; molecular graphics: *SHELXTL*; software used to prepare material for publication: *SHELXTL* and *PLATON* (Spek, 2009 ▶).

## Supplementary Material

Crystal structure: contains datablock(s) global, I. DOI: 10.1107/S1600536811023282/rz2615sup1.cif

Structure factors: contains datablock(s) I. DOI: 10.1107/S1600536811023282/rz2615Isup2.hkl

Supplementary material file. DOI: 10.1107/S1600536811023282/rz2615Isup3.cml

Additional supplementary materials:  crystallographic information; 3D view; checkCIF report

## Figures and Tables

**Table 1:** Hydrogen-bond geometry (Å, °) *Cg*2 and *Cg*3 are the centroids of the C20–C25 and C13–C18 rings, respectively.

*D*—H⋯*A*	*D*—H	H⋯*A*	*D*⋯*A*	*D*—H⋯*A*
C12—H12*A*⋯O1^i^	0.95	2.27	3.2019 (11)	167
C15—H15*A*⋯*Cg*2^ii^	0.95	2.81	3.6171 (9)	143
C2—H2*A*⋯*Cg*3^iii^	0.95	2.63	3.4304 (9)	143

Symmetry codes: (i) 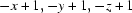; (ii) 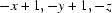; (iii) 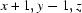.

**Fig. 1.**
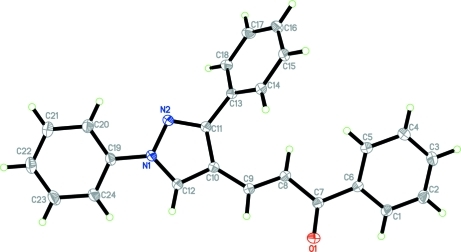
The asymmetric unit of the title compound, showing 30% probability displacement ellipsoids.

**Fig. 2.**
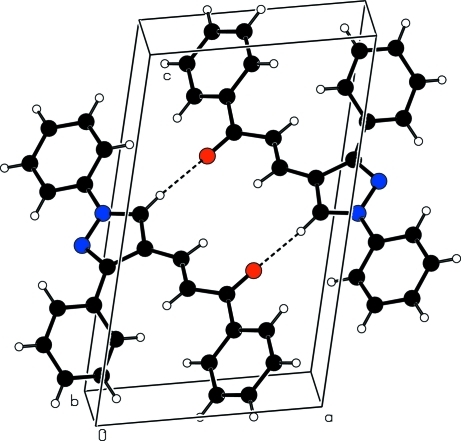
The crystal packing of the title compound. Intermolecular hydrogen bonds are shown as dashed lines.

## supplementary crystallographic information

### Comment

Pyrazoles are a novel class of heterocyclic compounds possessing wide variety of applications in the agrochemical and pharmaceutical industries (Patel *et al.*, 2004). Derivatives of pyrazoles are found to show good antibacterial (Isloor *et al.*, 2009; Vijesh *et al.*, 2010), anti-inflammatory (Sharma *et al.*, 2010), analgesic (Rostom *et al.*, 2003), anticancer, radioprotective (Ghorab *et al.*, 2010) and anti-convulsant activities (Amnekar *et al.*, 2010). Prompted by these diverse activities of pyrazole derivatives, we have synthesized the title compound to study its crystal structure.

In the title compound (Fig. 1), the pyrazole (N1/N2/C10–C12) group is essentially planar, with a maximum deviation of  0.004 (1) Å for atom C12, and makes dihedral angles of 18.07 (4)°, 48.60 (4)° and 9.13 (5)° with the adjacent C1–C6, C13–C18) and C19–C24 phenyl rings, respectively.

In the crystal structure (Fig. 2), adjacent molecules are connected *via* intermolecular C12—H12A···O1 (Table 1) hydrogen bonds forming dimers. Furthermore, the crystal structure is stabilized by weak π–π interactions between the pyrazole (N1/N2/C10–C12) and phenyl (C19–C24) rings [Cg···Cg = 3.6808 (5) Å; -x, 2-y, 1-z] and C—H···π (Table 1) interactions, involving the centroids of the C20–C25 (Cg2) and C13–C18 (Cg3) rings.

### Experimental

To a cold stirred mixture of methanol (20 ml) and sodium hydroxide (12.09 mmol) was added acetophenone (4.03 mmol). The reaction mixture was stirred for 10 min. To this solution was added formyl pyrazole (4.03 mmol) followed by tetrahydrofuran (30 ml). The solution was further stirred for 2 h at 0 °C and then at room temperature for 5 h. It was then poured into ice cold water. The resulting solution was neutralized with diluted HCl. The solid that separated out was filtered, washed with water, dried and crystallized from ethanol. Yield: 1.15 g, 81.5 %. M. p.: 406–408 K (Sharma *et al.*, 2010).

### Refinement

All hydrogen atoms were positioned geometrically [C–H = 0.95 Å] and were refined using a riding model, with *U*_iso_(H) = 1.2 *U*_eq_(C).

### Crystal data

**Table:**

C_24_H_18_N_2_O	*Z* = 2
*M**_r_* = 350.40	*F*(000) = 368
Triclinic, *P*1	*D*_x_ = 1.312 Mg m^−^^3^
Hall symbol: -P 1	Mo *K*α radiation, λ = 0.71073 Å
*a* = 8.1027 (2) Å	Cell parameters from 9964 reflections
*b* = 9.3157 (2) Å	θ = 2.3–34.3°
*c* = 12.9634 (3) Å	µ = 0.08 mm^−^^1^
α = 73.630 (1)°	*T* = 100 K
β = 74.713 (1)°	Block, colourless
γ = 74.820 (1)°	0.66 × 0.23 × 0.16 mm
*V* = 886.83 (4) Å^3^	

### Data collection

**Table:**

Bruker SMART APEXII CCD area-detector diffractometer	7371 independent reflections
Radiation source: fine-focus sealed tube	6190 reflections with *I* > 2σ(*I*)
graphite	*R*_int_ = 0.024
φ and ω scans	θ_max_ = 34.4°, θ_min_ = 1.7°
Absorption correction: multi-scan (*SADABS*; Bruker, 2009)	*h* = −12→12
*T*_min_ = 0.949, *T*_max_ = 0.988	*k* = −14→14
27273 measured reflections	*l* = −20→19

### Refinement

**Table:**

Refinement on *F*^2^	Primary atom site location: structure-invariant direct methods
Least-squares matrix: full	Secondary atom site location: difference Fourier map
*R*[*F*^2^ > 2σ(*F*^2^)] = 0.044	Hydrogen site location: inferred from neighbouring sites
*wR*(*F*^2^) = 0.131	H-atom parameters constrained
*S* = 1.04	*w* = 1/[σ^2^(*F*_o_^2^) + (0.0751*P*)^2^ + 0.1576*P*] where *P* = (*F*_o_^2^ + 2*F*_c_^2^)/3
7371 reflections	(Δ/σ)_max_ = 0.001
244 parameters	Δρ_max_ = 0.50 e Å^−^^3^
0 restraints	Δρ_min_ = −0.30 e Å^−^^3^

### Special details

**Table:**

Experimental. The crystal was placed in the cold stream of an Oxford Cryosystems Cobra open-flow nitrogen cryostat (Cosier & Glazer, 1986) operating at 100.0 (1) K.
Geometry. All esds (except the esd in the dihedral angle between two l.s. planes) are estimated using the full covariance matrix. The cell esds are taken into account individually in the estimation of esds in distances, angles and torsion angles; correlations between esds in cell parameters are only used when they are defined by crystal symmetry. An approximate (isotropic) treatment of cell esds is used for estimating esds involving l.s. planes.
Refinement. Refinement of F^2^ against ALL reflections. The weighted R-factor wR and goodness of fit S are based on F^2^, conventional R-factors R are based on F, with F set to zero for negative F^2^. The threshold expression of F^2^ > 2sigma(F^2^) is used only for calculating R-factors(gt) etc. and is not relevant to the choice of reflections for refinement. R-factors based on F^2^ are statistically about twice as large as those based on F, and R- factors based on ALL data will be even larger.

### Fractional atomic coordinates and isotropic or equivalent isotropic displacement parameters (Å^2^)

**Table:**

	*x*	*y*	*z*	*U*_iso_*/*U*_eq_	
O1	0.62674 (8)	0.29923 (7)	0.35790 (5)	0.02264 (13)	
N1	−0.05079 (9)	0.77669 (8)	0.50245 (5)	0.01568 (12)	
N2	−0.12324 (9)	0.77040 (8)	0.42026 (5)	0.01623 (12)	
C1	0.70994 (10)	0.10968 (9)	0.21010 (6)	0.01851 (14)	
H1A	0.7701	0.0780	0.2690	0.022*	
C2	0.75836 (11)	0.02845 (9)	0.12812 (7)	0.02144 (15)	
H2A	0.8514	−0.0584	0.1312	0.026*	
C3	0.67086 (12)	0.07414 (10)	0.04162 (7)	0.02281 (16)	
H3A	0.7029	0.0177	−0.0137	0.027*	
C4	0.53661 (12)	0.20236 (11)	0.03622 (7)	0.02424 (16)	
H4A	0.4781	0.2345	−0.0235	0.029*	
C5	0.48746 (11)	0.28408 (10)	0.11820 (7)	0.02067 (15)	
H5A	0.3953	0.3716	0.1142	0.025*	
C6	0.57315 (10)	0.23783 (8)	0.20632 (6)	0.01585 (13)	
C7	0.52409 (10)	0.31976 (8)	0.29762 (6)	0.01587 (13)	
C8	0.35228 (10)	0.42396 (9)	0.31234 (6)	0.01641 (13)	
H8A	0.2708	0.4290	0.2698	0.020*	
C9	0.31000 (10)	0.51223 (9)	0.38577 (6)	0.01634 (13)	
H9A	0.3974	0.5030	0.4251	0.020*	
C10	0.14917 (10)	0.61871 (8)	0.41212 (6)	0.01493 (12)	
C11	−0.00317 (10)	0.67559 (8)	0.36519 (6)	0.01476 (12)	
C12	0.11129 (10)	0.68879 (8)	0.49939 (6)	0.01588 (13)	
H12A	0.1858	0.6770	0.5481	0.019*	
C13	−0.04028 (10)	0.65220 (8)	0.26639 (6)	0.01507 (12)	
C14	0.08119 (10)	0.67017 (9)	0.16654 (6)	0.01730 (13)	
H14A	0.1920	0.6890	0.1640	0.021*	
C15	0.04095 (11)	0.66063 (9)	0.07101 (6)	0.02009 (14)	
H15A	0.1240	0.6731	0.0037	0.024*	
C16	−0.12143 (12)	0.63272 (10)	0.07439 (7)	0.02177 (15)	
H16A	−0.1498	0.6271	0.0092	0.026*	
C17	−0.24183 (11)	0.61317 (9)	0.17338 (7)	0.02090 (15)	
H17A	−0.3518	0.5928	0.1758	0.025*	
C18	−0.20240 (10)	0.62324 (9)	0.26905 (6)	0.01804 (14)	
H18A	−0.2858	0.6104	0.3362	0.022*	
C19	−0.14395 (10)	0.87250 (9)	0.57610 (6)	0.01661 (13)	
C20	−0.29759 (11)	0.97422 (9)	0.55476 (7)	0.01987 (14)	
H20A	−0.3394	0.9800	0.4915	0.024*	
C21	−0.38932 (12)	1.06744 (10)	0.62720 (7)	0.02283 (16)	
H21A	−0.4949	1.1362	0.6136	0.027*	
C22	−0.32750 (12)	1.06055 (10)	0.71912 (8)	0.02431 (17)	
H22A	−0.3906	1.1241	0.7684	0.029*	
C23	−0.17285 (12)	0.96010 (12)	0.73845 (8)	0.02685 (18)	
H23A	−0.1295	0.9565	0.8007	0.032*	
C24	−0.08031 (11)	0.86448 (11)	0.66779 (7)	0.02333 (16)	
H24A	0.0245	0.7949	0.6820	0.028*	

### Atomic displacement parameters (Å^2^)

**Table:**

	*U*^11^	*U*^22^	*U*^33^	*U*^12^	*U*^13^	*U*^23^
O1	0.0202 (3)	0.0266 (3)	0.0242 (3)	0.0010 (2)	−0.0109 (2)	−0.0099 (2)
N1	0.0156 (3)	0.0183 (3)	0.0144 (3)	−0.0022 (2)	−0.0034 (2)	−0.0065 (2)
N2	0.0160 (3)	0.0189 (3)	0.0151 (3)	−0.0022 (2)	−0.0045 (2)	−0.0059 (2)
C1	0.0177 (3)	0.0176 (3)	0.0184 (3)	0.0004 (2)	−0.0047 (2)	−0.0039 (2)
C2	0.0221 (4)	0.0182 (3)	0.0214 (3)	0.0009 (3)	−0.0033 (3)	−0.0062 (3)
C3	0.0245 (4)	0.0245 (4)	0.0203 (3)	−0.0032 (3)	−0.0022 (3)	−0.0103 (3)
C4	0.0233 (4)	0.0310 (4)	0.0191 (3)	−0.0001 (3)	−0.0069 (3)	−0.0093 (3)
C5	0.0184 (3)	0.0238 (4)	0.0193 (3)	0.0014 (3)	−0.0069 (3)	−0.0066 (3)
C6	0.0149 (3)	0.0161 (3)	0.0163 (3)	−0.0012 (2)	−0.0042 (2)	−0.0042 (2)
C7	0.0152 (3)	0.0157 (3)	0.0169 (3)	−0.0017 (2)	−0.0048 (2)	−0.0040 (2)
C8	0.0144 (3)	0.0171 (3)	0.0183 (3)	−0.0010 (2)	−0.0046 (2)	−0.0056 (2)
C9	0.0148 (3)	0.0180 (3)	0.0166 (3)	−0.0020 (2)	−0.0041 (2)	−0.0050 (2)
C10	0.0144 (3)	0.0158 (3)	0.0152 (3)	−0.0024 (2)	−0.0035 (2)	−0.0046 (2)
C11	0.0151 (3)	0.0156 (3)	0.0140 (3)	−0.0028 (2)	−0.0035 (2)	−0.0036 (2)
C12	0.0152 (3)	0.0180 (3)	0.0153 (3)	−0.0025 (2)	−0.0036 (2)	−0.0054 (2)
C13	0.0157 (3)	0.0149 (3)	0.0151 (3)	−0.0014 (2)	−0.0049 (2)	−0.0042 (2)
C14	0.0170 (3)	0.0189 (3)	0.0161 (3)	−0.0016 (2)	−0.0040 (2)	−0.0054 (2)
C15	0.0225 (3)	0.0214 (3)	0.0162 (3)	0.0002 (3)	−0.0047 (3)	−0.0076 (3)
C16	0.0265 (4)	0.0213 (3)	0.0207 (3)	−0.0010 (3)	−0.0099 (3)	−0.0087 (3)
C17	0.0226 (4)	0.0206 (3)	0.0231 (4)	−0.0050 (3)	−0.0104 (3)	−0.0048 (3)
C18	0.0180 (3)	0.0190 (3)	0.0182 (3)	−0.0039 (2)	−0.0060 (3)	−0.0037 (2)
C19	0.0165 (3)	0.0183 (3)	0.0160 (3)	−0.0045 (2)	−0.0007 (2)	−0.0070 (2)
C20	0.0200 (3)	0.0191 (3)	0.0196 (3)	−0.0020 (3)	−0.0025 (3)	−0.0065 (3)
C21	0.0220 (4)	0.0198 (3)	0.0249 (4)	−0.0023 (3)	0.0004 (3)	−0.0089 (3)
C22	0.0237 (4)	0.0251 (4)	0.0257 (4)	−0.0073 (3)	0.0036 (3)	−0.0140 (3)
C23	0.0239 (4)	0.0377 (5)	0.0240 (4)	−0.0064 (3)	−0.0012 (3)	−0.0182 (3)
C24	0.0198 (4)	0.0321 (4)	0.0212 (4)	−0.0022 (3)	−0.0038 (3)	−0.0143 (3)

### Geometric parameters (Å, °)

**Table:**

O1—C7	1.2305 (9)	C11—C13	1.4742 (10)
N1—C12	1.3508 (10)	C12—H12A	0.9500
N1—N2	1.3661 (8)	C13—C18	1.3985 (11)
N1—C19	1.4232 (9)	C13—C14	1.4016 (11)
N2—C11	1.3325 (10)	C14—C15	1.3921 (10)
C1—C2	1.3906 (11)	C14—H14A	0.9500
C1—C6	1.3995 (11)	C15—C16	1.3939 (12)
C1—H1A	0.9500	C15—H15A	0.9500
C2—C3	1.3904 (12)	C16—C17	1.3903 (13)
C2—H2A	0.9500	C16—H16A	0.9500
C3—C4	1.3883 (13)	C17—C18	1.3926 (11)
C3—H3A	0.9500	C17—H17A	0.9500
C4—C5	1.3935 (11)	C18—H18A	0.9500
C4—H4A	0.9500	C19—C24	1.3918 (11)
C5—C6	1.3989 (11)	C19—C20	1.3930 (11)
C5—H5A	0.9500	C20—C21	1.3936 (11)
C6—C7	1.4978 (10)	C20—H20A	0.9500
C7—C8	1.4742 (11)	C21—C22	1.3892 (13)
C8—C9	1.3496 (10)	C21—H21A	0.9500
C8—H8A	0.9500	C22—C23	1.3885 (14)
C9—C10	1.4416 (10)	C22—H22A	0.9500
C9—H9A	0.9500	C23—C24	1.3937 (12)
C10—C12	1.3886 (10)	C23—H23A	0.9500
C10—C11	1.4305 (10)	C24—H24A	0.9500
			
C12—N1—N2	111.99 (6)	N1—C12—H12A	126.2
C12—N1—C19	127.99 (6)	C10—C12—H12A	126.2
N2—N1—C19	119.98 (6)	C18—C13—C14	119.08 (7)
C11—N2—N1	105.17 (6)	C18—C13—C11	120.35 (7)
C2—C1—C6	120.43 (7)	C14—C13—C11	120.39 (7)
C2—C1—H1A	119.8	C15—C14—C13	120.62 (7)
C6—C1—H1A	119.8	C15—C14—H14A	119.7
C3—C2—C1	120.10 (7)	C13—C14—H14A	119.7
C3—C2—H2A	119.9	C14—C15—C16	119.86 (8)
C1—C2—H2A	119.9	C14—C15—H15A	120.1
C4—C3—C2	119.96 (7)	C16—C15—H15A	120.1
C4—C3—H3A	120.0	C17—C16—C15	119.82 (7)
C2—C3—H3A	120.0	C17—C16—H16A	120.1
C3—C4—C5	120.16 (8)	C15—C16—H16A	120.1
C3—C4—H4A	119.9	C16—C17—C18	120.51 (7)
C5—C4—H4A	119.9	C16—C17—H17A	119.7
C4—C5—C6	120.30 (7)	C18—C17—H17A	119.7
C4—C5—H5A	119.8	C17—C18—C13	120.11 (7)
C6—C5—H5A	119.8	C17—C18—H18A	119.9
C5—C6—C1	119.04 (7)	C13—C18—H18A	119.9
C5—C6—C7	122.63 (7)	C24—C19—C20	120.83 (7)
C1—C6—C7	118.34 (6)	C24—C19—N1	119.87 (7)
O1—C7—C8	121.76 (7)	C20—C19—N1	119.30 (7)
O1—C7—C6	119.81 (7)	C19—C20—C21	119.29 (8)
C8—C7—C6	118.43 (6)	C19—C20—H20A	120.4
C9—C8—C7	120.44 (7)	C21—C20—H20A	120.4
C9—C8—H8A	119.8	C22—C21—C20	120.50 (8)
C7—C8—H8A	119.8	C22—C21—H21A	119.7
C8—C9—C10	128.27 (7)	C20—C21—H21A	119.7
C8—C9—H9A	115.9	C23—C22—C21	119.52 (8)
C10—C9—H9A	115.9	C23—C22—H22A	120.2
C12—C10—C11	103.95 (6)	C21—C22—H22A	120.2
C12—C10—C9	123.14 (7)	C22—C23—C24	120.88 (8)
C11—C10—C9	132.89 (7)	C22—C23—H23A	119.6
N2—C11—C10	111.32 (6)	C24—C23—H23A	119.6
N2—C11—C13	117.90 (6)	C19—C24—C23	118.96 (8)
C10—C11—C13	130.69 (7)	C19—C24—H24A	120.5
N1—C12—C10	107.56 (6)	C23—C24—H24A	120.5
			
C12—N1—N2—C11	0.28 (8)	C11—C10—C12—N1	0.79 (8)
C19—N1—N2—C11	178.30 (7)	C9—C10—C12—N1	−178.05 (7)
C6—C1—C2—C3	−0.03 (13)	N2—C11—C13—C18	−47.06 (10)
C1—C2—C3—C4	0.94 (13)	C10—C11—C13—C18	136.80 (8)
C2—C3—C4—C5	−1.00 (14)	N2—C11—C13—C14	128.07 (8)
C3—C4—C5—C6	0.15 (14)	C10—C11—C13—C14	−48.06 (11)
C4—C5—C6—C1	0.76 (12)	C18—C13—C14—C15	0.52 (11)
C4—C5—C6—C7	−179.03 (8)	C11—C13—C14—C15	−174.68 (7)
C2—C1—C6—C5	−0.82 (12)	C13—C14—C15—C16	−0.10 (12)
C2—C1—C6—C7	178.98 (7)	C14—C15—C16—C17	−0.59 (12)
C5—C6—C7—O1	−162.50 (8)	C15—C16—C17—C18	0.85 (12)
C1—C6—C7—O1	17.72 (11)	C16—C17—C18—C13	−0.43 (12)
C5—C6—C7—C8	17.37 (11)	C14—C13—C18—C17	−0.26 (11)
C1—C6—C7—C8	−162.41 (7)	C11—C13—C18—C17	174.95 (7)
O1—C7—C8—C9	6.89 (12)	C12—N1—C19—C24	−10.42 (12)
C6—C7—C8—C9	−172.98 (7)	N2—N1—C19—C24	171.91 (7)
C7—C8—C9—C10	−179.26 (7)	C12—N1—C19—C20	169.10 (8)
C8—C9—C10—C12	171.25 (8)	N2—N1—C19—C20	−8.57 (11)
C8—C9—C10—C11	−7.21 (14)	C24—C19—C20—C21	−0.83 (12)
N1—N2—C11—C10	0.25 (8)	N1—C19—C20—C21	179.65 (7)
N1—N2—C11—C13	−176.60 (6)	C19—C20—C21—C22	0.74 (13)
C12—C10—C11—N2	−0.66 (9)	C20—C21—C22—C23	0.16 (13)
C9—C10—C11—N2	178.01 (8)	C21—C22—C23—C24	−1.00 (14)
C12—C10—C11—C13	175.68 (8)	C20—C19—C24—C23	0.02 (13)
C9—C10—C11—C13	−5.65 (14)	N1—C19—C24—C23	179.53 (8)
N2—N1—C12—C10	−0.70 (9)	C22—C23—C24—C19	0.91 (14)
C19—N1—C12—C10	−178.53 (7)		

### Hydrogen-bond geometry (Å, °)

**Table:**

Cg2 and Cg3 are the centroids of the C20–C25 and C13–C18 rings, respectively.

**Table:**

*D*—H···*A*	*D*—H	H···*A*	*D*···*A*	*D*—H···*A*
C12—H12A···O1^i^	0.95	2.27	3.2019 (11)	167
C15—H15A···Cg2^ii^	0.95	2.81	3.6171 (9)	143
C2—H2A···Cg3^iii^	0.95	2.63	3.4304 (9)	143

Symmetry codes: (i) −*x*+1, −*y*+1, −*z*+1; (ii) −*x*+1, −*y*+1, −*z*; (iii) *x*+1, *y*−1, *z*.
